# Quantitative analysis of *Staphylococcus aureus* in patients with chronic rhinosinusitis under continuous ultrasound treatment

**Published:** 2018-12

**Authors:** Narjes Feizabadi, Javad Sarrafzadeh, Mojtaba Fathali, Behnoosh Vasaghi-Gharamaleki, Mahdi Dadgoo, Hossein Kazemian, Jalil Kardan-Yamchi, Sonia Hesam Shariati

**Affiliations:** 1 Department of Physiotherapy, School of Rehabilitation, Iran University of Medical Sciences, Tehran, Iran; 2 Surgeon and Specialist in Ear, Nose and Troat, School of Rehabilitation, Tehran University of Medical Sciences, Tehran, Iran; 3 Department of Basic Sciences, School of Rehabilitation, Iran University of Medical Sciences, Tehran, Iran; 4 Clinical Microbiology Research Center, Ilam University of Medical Sciences, Ilam, Iran; 5 Department of Medical Microbiology, School of Medicine, Tehran University of Medical Sciences, Tehran, Iran; 6 Department of Pathobiology, School of Public Health, Tehran University of Medical Sciences, Tehran, Iran

**Keywords:** Chronic rhinosinusitis, Ultrasound treatment, *Staphylococcus aureus*

## Abstract

**Background and Objectives:**

Bacterial pathogens, in particular drug resistant strains, involved in chronic rhinosinusitis may result in treatment failure. Ultrasound waves are able to destroy bacterial population in sinus cavities and can recover patients.

**Materials and Methods:**

Twelve patients with chronic sinusitis and 10 healthy controls were treated by continuous ultrasound waves. Clinical specimens were collected before and after treatment. Serial diluted specimens were cultured on blood agar, chocolate and MacConkey agar plates for bacterial isolation. Bacterial DNA was extracted and used for *Staphylococcus aureus* detection using quantitative PCR.

**Results:**

*S. aureus* was the most isolated bacterium (10 patients), which was eradicated from 8 patients after treatment. Using phenotypic methods at the beginning, 3 out of 10 healthy individuals were found to be positive. From 11 positive patients for *S. aureus* identified by real time qPCR, 9 showed significant reduction after treatment. In the healthy group, *S. aureus* was detected in 4 samples using qPCR, but they were clean at the second sampling.

**Conclusion:**

According to our phenotypic and molecular experiments, continuous ultrasound treatment effectively reduced the bacterial population in studied patients (p < 0.01). This was a hopeful basis for doing more studies with ultrasound therapy as a treatment option.

## INTRODUCTION

Chronic rhinosinusitis (CRS) is a chronic inflammation of the sinonasal mucosa with multifactorial causes (1). Many factors, such as mucosal inflammation, mucociliary dysfunction, and microbial conditions, contribute to this disease (2). Among these factors, bacterial agents play an important role in CRS. It has been reported that patients with CRS appear to have an increased abundance of certain bacteria species, especially *Staphylococcus aureus* (3, 4).

Bacteria can form biofilms on the paranasal epithelium in refractory sinus patients in spite of repetitive treatment with antibiotics (5). A biofilm is a polymicrobial community with altered metabolism, growth, and gene transcription phenotypes. This community is embedded in a self-produced extracellular matrix which let the bacteria to attach to surfaces and resist to harsh conditions (6). The biofilms have been observed more frequently on the mucosa of patients with CRS relative to control groups (7).

Although CRS antibiotic therapy reduces bacterial population in short-term, it allows colonization with bacterial cells that are less susceptible to the prescribed antibiotics or biofilm producing bacteria. A biofilm state makes bacterial cells resistant to most antibiotics (3, 8). However, these biofilms responds to treatment if they expose to ultrasonic (US) waves *in vitro* (9–12).

Ultrasound therapyis capable of killing bacteria by disrupting the bacterial cell membrane (9, 13) and generation of free radicals, causing eventual bacterial death (9, 14). Ultrasound therapy can also damage the biofilm matrix and decrease the biofilm stability (15).

Because of the bacterial antibiotic resistance within biofilms, new treatment options and strategies are needed to overcome this therapeutic challenge. Therefore, the aim of this study was to investigate the effect of ultrasound treatment on *S. aureus*, which is one of the prevalent pathogens in patients with rhinosinusitis. Bacterial culture and quantitative real-time PCR were used to follow-up the fluctuations of this agent before and after US treatment.

## MATERIALS AND METHODS

### Study design.

This was a double-blind randomized clinical trial which was conducted from January 2018 to May 2018. The study population included adult patients with the following criteria: clinical diagnostic criteria of more than or equal to 2 major symptoms or 1 major symptom, including nasal obstruction, facial pain/pressure, postnasal drip, and hyposmia; and 2 minor symptoms, including headache, halitosis, fatigue, dental pain, and ear pain); and CT scan findings of patients with CRS who had failed medical treatment or surgery. Patients with head and neck malignancy or those who had a current medical disease for which US was contraindicated were excluded. All patients were recruited and referred by an ear, nose, and throat (ENT) surgeon. In total, 12 patients with CRS and 10 healthy people who voluntarily filled out the informed consent form were included in this study.

### Specimen collection.

All participants were subjected to sampling for microbiological analysis and imaging CT scan 2 times, with a 3-week interval. Specimen collection was done before and after treatment for patients and for the first and second time for healthy controls without any intervention. Samples were taken from the right and left meatus and nasopharynx by Dacron swabs. Then, swabs transferred to laboratory in sterile tubes, including 1 mL saline solution for further analysis. Also, SNOT-20 questionnaire was filled 2 times before and after the treatment (supplementary data).

### Ultrasound treatment.

All patients were treated by continuous US, with the probe velocity movement of 4 cm/s. The intensity was 1 W/cm^2^ for maxillary sinuses, and it was 0.5 W/cm^2^ for frontal sinuses. Treatment period was 10 sessions intermittently during 3 weeks. After the treatment period, sampling was repeated for microbiological analysis and CT scan imaging. If CT scan did not show recovery, routine treatment procedure started for the patient by an ENT physician.

### Laboratory bacterial identification and colony-forming unit calculation.

All samples were transferred immediately to microbiology laboratory of Tehran University of Medical Sciences in 10 mL falcon tubes containing 1 mL saline solution. Each falcon was vortexed before culturing. Then, 10 microliter of each sample was serially diluted from 10^1^ to 10^−6^ concentration in PBS. Finally, 10 microliter of each concentration was separately cultured by spread culture method in 2 sheep blood agar, 2 chocolate agar and MacConkey agar. Cultures media were incubated aerobically and anaerobically at 37°C for 24 hours. The colony-forming units (CFUs)/mL of each sample was calculated after 24 hours using Miles and Misra Method (16). After calculating CFU, all the suspected bacterial grown colonies were identified by Gram staining, a standard routine biochemical and microbiological test according to Mahon et al. (17).

### Primers and probe.

The fibronectin-binding protein A (FnbA) gene was used to detect *S. aureus* in patients's pecimens. The sequences of primers and probe used for amplification of *fnbA* gene is shown in Table 1 (18).

**Table 1.** Primers and probe for *fnbA* gene

	**Sequence**	**Tm°C**	**Product size**
Forward primer	5-AGTGAGCGACCATACAACAG-3	58.4	
Reverse primer	5-CATAATTCCCGTGACCATTT-3	54.3	185 bp
Probe	5-FAM-AAGCACAAGGACCAATCGAGG-BHQ-1-3	61.2	

### DNA extraction.

Bacterial DNA was extracted from *S. aureus* ATCC 29213 strain and clinical specimens using Macherey Nagel (MN), a DNA purification kit (Macherey Nagel, Germany). First, 1 mL of collecting sample was centrifuged for 10 minutes at 13000 rpm. The pellet was suspended in 200 uL of 1XTE buffer with 0.04 mg/mL of lysozyme. Following steps were done according to the manufacturer protocol (Macherey Nagel, Germany). Finally, extracted DNA was eluted in 50 uL elution buffer and saved in −20°C for further procedures.

### Quantitative real-time PCR.

DNA from *S. aureus* ATCC 29213 was used as a positive control. The concentrations of DNA templates were determined using Nanodrop 1000 (Thermo Scientific, USA). The following formula was used to calculate the molecules of template per gram: molecules of DNA = mass (in gram) Avogadro's number/average molecular weight of a base × template length (19). To gain the efficacy of real-time PCR, a 10-fold serial dilutions of DNA was used for the standard curve of assay.

LinGene K Real-Time PCR apparatus (Bioer, Hangzhou, China) was used for real-time PCR assays. Each reaction contained 1 uL of 10 pM forward and reverse primers, 0.1 mM of TaqMan probe, 1 uL of template, in addition to qPCR Master mix (Amplicon, Danmark), and distilled water in a total volume of 25 uL. Real-time PCR was run as follows: 95°C for 5 minutes, 95°C for 20 seconds, 59°C for 20 seconds, and 72°C for 40 seconds, repeating 50 cycles of steps 2 to 4. All the samples were run in duplicate.

### Data analysis.

Chi-square test was used to compare the presence of *S. aureus* among patients and control groups. Also, addition nonparametric McNemar's test was used to determine phenotypically *S. aureus* presence in samples before and after the treatment. The Wilcoxon test was used to analyze our qPCR results between related groups before and after the treatment. This was done by SPSS software version 21. A p-value of <0.05 was determined as statistically significant.

## RESULTS

### Ultrasound treatment outcome.

**Fig. 1.**
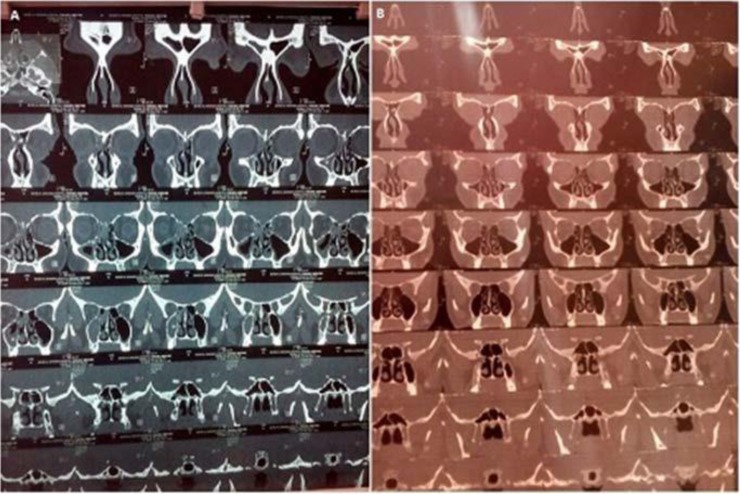
CT scan findings before (A) and after (B) the treatment

CT findings showed a remarkable reduction after the treatment for 8 out of 12 patients. The 4 remained patients had the same score before and after the treatment (Fig. 1, Table 2). In addition, the questionnaire results demonstrated a notable reduction of the all patient scores after treatment. Both of these results represent that US treatment was significantly successful in the patient's recovery (Table 2).

**Table 2.** CT and SNOT-20 questionnaire scores of patients before and after the treatment.

**Patient ID**	**CT scores**		**Questionnaire scores**	

	**Before**	**After**	**Before**	**After**
1	17	17	40	5
2	6	6	36	25
3	6	6	43	23
4	13	13	25	5
5	20	12	34	17
6	18	0	36	6
7	25	0	49	9
8	8	0	76	18
9	36	0	74	48
10	29	0	43	7
11	30	1	22	5
12	25	0	48	5

### Phenotypical analysis.

Using bacteriology method, more *S. aureus* was detected in patients (n=10) than in healthy control group (n=3) (p-value= 0.011). In addition to *S. aureus*, other pathogenic and non-pathogenic bacteria, such as *Streptococcus pneumoniae, Haemophilus influenzae, Klebsiella pneumoniae, Enterobacter cloacae, Enterobacteriaceae* family, nonhemolytic streptococci, Gram-negative cocci and coagulase-negative staphylococci were also identified. After treatment, *S. aureus* was eradicated from 8 patients (p-value= 0.011).

### Real-time qPCR output.

According to the quantitative PCR results, 4 out of 10 individuals in the healthy control group were positive for *S. aureus* on the first sampling, but they were all negative at the second time. In the patient group, *S. aureus* was not detected in 1 patient, but all others showed different copies of examining gene as shown in Table 3. All the positive patients showed significant reduction (p < 0.01) for studied agent, except for 2 of them. *S. aureus* has been slightly increased in nasopharynx of 1 patient (No. 8) and left meatus of another patient (No. 4) was positive after treatment while it was clear before treatment.

**Table 3.** Copy number of *fnbA* gene in patients group.

**Patient ID**	**Site of sampling**	**Before treatment (mean values)**	**Before treatment (SD)**	**After treatment (mean values)**	**After treatment (SD)**
1	Nasopharynx	29100	192.3	N^*^	-
	Rightmeatus	N	-	N	-
	Leftmeatus	N	-	N	-
2	Nasopharynx	900.2	48.4	275	18.4
	Rightmeatus	269	12.7	N	-
	Leftmeatus	20900	113.1	N	-
3	Nasopharynx	7440	63.6	283	18.4
	Rightmeatus	6290	43.8	1440	56.5
	Leftmeatus	N	-	N	-
4	Nasopharynx	N	-	N	-
	Rightmeatus	N	-	N	-
	Leftmeatus	N	-	7190	155.5
5	Nasopharynx	N	-	N	-
	Rightmeatus	11300	91.9	N	-
	Leftmeatus	81000	169.7	N	-
6	Nasopharynx	N	-	N	-
	Rightmeatus	N	-	N	-
	Leftmeatus	166	8.5	N	-
7	Nasopharynx	14700	90.5	N	-
	Rightmeatus	15900	183.8	N	-
	Leftmeatus	8550	70.7	N	-
8	Nasopharynx	28.3	4.1	126	16.6
	Rightmeatus	468000	670.5	24600	219.2
	Leftmeatus	513	24	60.5	7.6
9	Nasopharynx	N	-	N	-
	Rightmeatus	289	19.8	N	-
	Leftmeatus	N	-	N	-
10	Nasopharynx	N	-	N	-
	Rightmeatus	99.8	13.3	N	-
	Leftmeatus	497	26.9	N	-
11	Nasopharynx	N	-	N	-
	Rightmeatus	372	25.4	N	-
	Leftmeatus	18600	182.4	N	-
12	Nasopharynx	N	-	N	-
	Rightmeatus	N	-	N	-
	Leftmeatus	N	-	N	-

* N; negative

## DISCUSSION

It has been reported that many bacteria residing on mucosal surfaces of patients with CRS exist in a biofilm state, making them resistant to most systemic antibiotics (7). Unlike relative success of treatment of these patients, antibiotics are the most common choice for CRS condition (20, 21). Moreover, the widespread use of antibiotics seems to result in development of resistant bacteria, and thus treatment failure (22). Bhattacharyya and Kepnes showed increased methicillin resistant *S. aureus* (MRSA) in CRS conditions due to extensive encounter of sinus microbiome to the antibiotics (22). *S. aureus* is one of the important agents which can produce the thick layer of biofilm (23). Hence, perhaps this factor makes *S. aureus* an important colonizer of CRS patients (3, 4). Increasing resistance against common antibiotic options for CRS treatment has convinced researchers to investigate alternative therapies (24). In theory, US is breaking down bacterial biofilms and make bacterial population reduction in CRS patients (25).

In the present study, the efficacy of US treatment targeting *S. aureus* populationin CRS patients was evaluated. According to the literature, *S. aureus* is the most frequent bacteria isolated from CRS patients (3, 4). Based on the results of this study, continuous US treatment reduced *S. aureus* considerably as determined by both phenotypic and genotypic methods. These results were also inconsistent with the CT and questionnaire scores. Results of qPCR showed significant differences among the number of organisms before and after the US treatment. All the *S. aureus* positive patients showed significant reduction after treating with US, except for 2 of them. This may cause by sample contamination or patient's secondary colonization by *S. aureus* during the process. However, this was the first report on the quantitative analysis of this organism in these patients and the results were in agreement with patients who responded to therapy (data not provided).

A study was conducted on 48 acute sinusitis patients (24 in the patient group and 24 in the control group). The control group received amoxicillin 500 mg 3 times a day for 10 days, while the patient group received 4 consecutive days of US. They showed relapses and they also found that future attack was reduced in patients who were treated by US (26). In another study 57 CRS patients were treated by low-intensity pulsed US (27). They reported that almost major and minor symptoms showed significant changes after US treatment. According to Naghdi et al. (28), 30 patients with CRS were treated by 10 time of continuous US. At the end of treatment, the “percent improvement” was 74%. Also, 72% of patients reported continued improvement 1 month after treatment. Our results were in accordance with those of Naghdi et al study. In addition to CRS patient's improvement, the *S. aureus* count was reduced dramatically after US therapy.

US method can potentially reduce the microorganism's mass in sinuses and can be helpful in patients'treatment. Nevertheless, our patient's population was limited and more patients are needed for future studies. Also, evaluation of other involved agents could provide useful information in this regard. This data can be used as a basis for next studies on similar patients under different treatment conditions and for analyzing more potential agents.
